# Older persons’ experiences of adapting to daily life at home after hospital discharge: a qualitative metasummary

**DOI:** 10.1186/s12913-019-4035-z

**Published:** 2019-04-11

**Authors:** Christine Hillestad Hestevik, Marianne Molin, Jonas Debesay, Astrid Bergland, Asta Bye

**Affiliations:** 1 Department of Physiotherapy, Faculty of Health Sciences, OsloMet - Oslo Metropolitan University, Oslo, Norway; 2 Department of Nursing and Health Promotion, Faculty of Health Sciences, OsloMet - Oslo Metropolitan University, Oslo, Norway; 3 Bjørknes University College, Lovisenberggata 13, 0456 Oslo, Norway; 4 European Palliative Care Research Centre (PRC), Department of Oncology, Oslo University Hospital and Institute of Clinical Medicine, University of Oslo, Oslo, Norway

**Keywords:** Transition, Older people, Informal caregiver, Patient involvement, Communication, Qualitative research, Metasummary

## Abstract

**Background:**

Researchers have shown that hospitalisation can decrease older persons’ ability to manage life at home after hospital discharge. Inadequate practices of discharge can be associated with adverse outcomes and an increased risk of readmission. This review systematically summarises qualitative findings portraying older persons’ experiences adapting to daily life at home after hospital discharge.

**Methods:**

A metasummary of qualitative findings using Sandelowski and Barroso’s method. Data from 13 studies are included, following specific selection criteria, and categorised into four main themes.

**Results:**

Four main themes emerged from the material: (1) Experiencing an insecure and unsafe transition, (2) settling into a new situation at home, (3) what would I do without my informal caregiver? and (4) experience of a paternalistic medical model.

**Conclusions:**

The results emphasise the importance of assessment and planning, information and education, preparation of the home environment, the involvement of the older person and caregivers and supporting self-management in the discharge and follow-up care processes at home. Better communication between older persons, hospital providers and home care providers is needed to improve the coordination of care and facilitate recovery at home. The organisational structure may need to be redefined and reorganised to secure continuity of care and the wellbeing of older persons in transitional care situations.

**Electronic supplementary material:**

The online version of this article (10.1186/s12913-019-4035-z) contains supplementary material, which is available to authorized users.

## Background

For older people, hospitalisation and changes in health status are often followed by feelings of stress, anxiety and uncertainty about the future [1]. Research has shown that hospitalisation decreases physical function, increases dependence [2] and decreases health-related quality of life (HRQOL). Older persons’ HRQOL has also been found to decline during the post-discharge period [3, 4]. After hospital discharge, these individuals tend to face many challenges to adjusting and coping with the possible repercussions of their illness (es) at home [5, 6].

According to surveys, older people generally want to stay in their own home for as long as possible [7, 8]. The policies of welfare states emphasise providing home care services with the goal of preserving the dignity and wellbeing of older people [7], and high-quality transitional care helps older people with multiple chronic conditions remain in their own homes for as long as possible. Additionally, it has the potential to minimise adverse events and rehospitalisation and increase the efficiency of the whole healthcare system [9, 10].

However, older persons face a myriad of challenges during this process. Multiple studies have reported that older persons experience a discontinuity of care on their way from hospital back into their community [11–14]. Shortened hospital stays and lack of continuity of care when older persons transition from hospital to home have been identified as serious challenges with negative implications, such as increased readmission rates and adverse medical events [15–17]. Furthermore, as Ekdahl et al. [18] stated, although older persons and geriatric syndromes are common in hospitals, these persons are commonly not prioritised, as healthcare professionals often perceive their cases as being too complex and time-consuming. Research shows that there is a lack of attention given to these persons’ special needs and inadequate involvement of them and their families in their own care process [12, 19–21]. Another problem is the inadequate communication of information between hospitals and other healthcare providers [22]. Bull et al. [23] found that the best predictors of older persons’ satisfaction with discharge planning were a perception of continuity of care and preparedness to manage their own care. Almborg et al. [24] suggested that if the patients were provided with information about how to evaluate symptoms, manage medication and restrict activities, they felt more prepared after discharge. Moreover, the study emphasised that healthcare providers evaluation of the patient’s needs after discharge is essential to the patient, and different professional disciplines should be involved depending on the patient’s conditions and needs [25]. The older person’s participation in the evaluation of their needs could be facilitated by asking them about problems in different areas of their life, among other strategies.

A key challenge of transitional care is providing healthcare adapted to the needs of older people—as perceived by themselves, not as defined by the professionals. Previous systematic reviews have found that transitional care interventions can be effective in improving outcomes [26–28]. However, challenges still remain in enhancing older persons’ satisfaction with the healthcare services included in transitional care [9, 29]. Knowledge about the experiences of older people regarding their own care is crucial to identifying and addressing issues related to the transition from hospital to home and may help reduce deficiencies and facilitate more satisfactory healthcare [30]. Thus, this metasummary aims to integrate current international findings in order to enhance the understanding of older persons’ experiences of adapting to daily life at home after hospital discharge.

## Methods

### Study design

The techniques used to conduct this metasummary followed the methodological framework of Sandelowski and Barroso [31]. Qualitative metasummary is a quantitatively-oriented aggregation approach to research synthesis. Qualitative findings are collected from topical or thematic surveys of the data through a review of the relevant literature. In a qualitative metasummary, higher frequency findings are taken in order to find evidence of the repetition imperative to validity in quantitative research and to having discovered a pattern or theme.

### Study retrieval—search strategy

With assistance from a librarian, the first author conducted a comprehensive literature review using five electronic databases (Medline, Embase, Academic Search Premier, Cinahl and PsycINFO). Hand searches were conducted and reference lists were examined. Keywords for the databases searches were: *Aged, older patient, frail, elderly linked with patient discharge, patient transfer, patient handover, transitional care, hospital to home, hospital to municipal, hospital to community, patient* (*satisfaction, perception, experience, perspective, view*) and *interview* or *focus groups*. The search was limited to studies published in the English language between 2006 and 2017 (current), aiming at findings that reflect patients’ experiences of up-to-date healthcare systems.

### Selection criteria

Titles, abstracts or full-text studies were scanned for adherence to the following inclusion criteria: studies using qualitative methods, a semi-structured or open-ended questioning approach; exploring older persons’ self-reported experiences with relevance to the research topic; experiences of persons aged 65 or over adapting to life at home after hospital discharge. Original research, including peer-reviewed articles and doctoral theses, were included.

The studies were individually appraised using the Johanna Briggs Institute Qualitative Assessment and Review Instrument (JBI-QARI) [32]. The purpose of this appraisal was to assure that the reports met the inclusion criteria and to familiarise the authors with the informational content, methodological orientation and style and form of each study [31]. A cross-study comparative appraisal was also conducted using the GRADE-CERQual approach [33]. This method involves displaying the same key elements of information in each report alongside each other to determine how the studies related and help explain and contextualise the findings in the reports [31]. Individual and comparative appraisals were discussed among the authors until an agreement was reached.

### Synthesis of findings

Data analysis in qualitative research consists of preparing and organising data (e.g. texts such as transcripts of interviews) for analysis and then reducing the data into broad patterns or themes [34]. The selected articles were reviewed, and relevant findings were extracted from each study, followed by grouping the findings into thematic statements and summarising these into abstracted themes. We calculated the frequency effect for each thematic statement by dividing the number of studies that mentioned a finding by the total number of studies included in the metasummary. This was done in order to quantify the strength of the findings, ensuring that the importance of these findings was neither neglected nor over-emphasised.

## Results

**Fig. 1:**
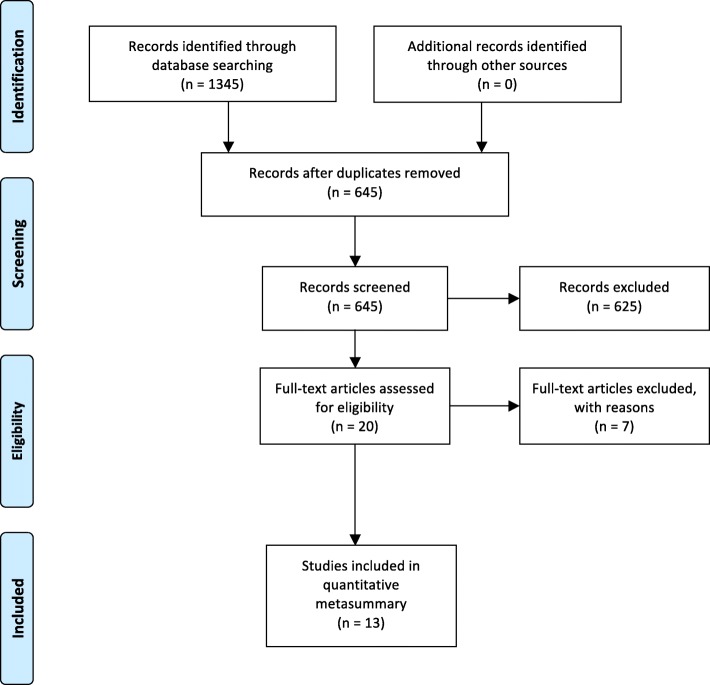
Identification and selection of studies. Source: Moher, Liberati, Tetzlaff, Altman, and The PRISMA Group. (2009). *Note.* For more information, visit www.prisma-statement.org

The initial search identified 1345 studies. After removing duplicates, we ended up with 645 studies. An additional 625 studies were excluded after screening the titles and abstracts, as they lacked relevance to the study’s topic. Twenty studies met inclusion criteria and were retrieved in full text for further analysis. Five studies were excluded because the findings were not relevant for the study’s topic, as well as an additional two since some of the participants were interviewed in nursing homes, which made it difficult to separate findings concerning their experiences from nursing homes and their own homes. To make sure that no relevant studies were omitted, we scrutinised the reference lists of the selected studies and relevant literature reviews [35, 36], but no additional studies were found. No studies were excluded on the basis of quality appraisal (Table 1). One of the studies explored the perceptions of older persons who were readmitted to hospital within 28 days of discharge [37]. We decided to include this study, as it portrayed valuable experiences of the discharge process, as well as experiences of settling at home after discharge. The final sample included 13 studies meeting the criteria for the metasummary (Fig. 1). Each study was systematically assessed for its research question or statement of purpose, research method, sample size, participant characteristics (age, sex and diagnosis), setting and country in which the research took place (Table 2).

**Table 1:** Quality assessment of the included studies using the JBI-QARI appraisal instrument

Questions	Andreasen et al	Bagge et al.	Dilworth et al.	Dossa et al.	Jones	Karlsson et al.	Knight et al.	McKeown et al	Neiterman et al.	Perry et al.	Reay et al.	Rydeman et al.	Slatyer et al.
Is there congruity between the stated philosophical perspective and the research methodology?	U	U	NA	U	Y	Y	U	Y	U	Y	Y	NA	Y
Is there congruity between the research methodology and the research question or objectives?	Y	Y	Y	Y	Y	Y	Y	Y	Y	Y	Y	Y	Y
Is there congruity between the research methodology and the methods used to collect data?	Y	Y	Y	Y	Y	U	Y	Y	Y	Y	Y	Y	Y
Is there congruity between the research methodology and the representation and analysis of data?	Y	Y	Y	Y	Y	Y	Y	Y	Y	Y	Y	Y	Y
Is there congruity between the research methodology and the interpretation of results?	Y	Y	Y	Y	Y	Y	N	Y	Y	Y	Y	Y	Y
Is there a statement that locates the researcher culturally or theoretically?	N	N	U	N	Y	N	N	Y	N	Y	N	N	N
Is the influence of the researcher on the research, and vice versa, addressed?	Y	Y	Y	Y	Y	Y	N	Y	N	N	U	Y	Y
Are participants, and their voices, adequately represented?	Y	Y	Y	Y	Y	Y	U	Y	Y	Y	Y	Y	Y
Is the research ethical according to current criteria or, for recent studies, is there evidence of ethical approval by an appropriate body?	Y	Y	Y	Y	Y	Y	Y	Y	Y	Y	Y	Y	Y
Do the conclusions drawn in the research report flow from the analysis, or interpretation, of the data?	Y	Y	Y	Y	Y	Y	Y	Y	Y	Y	Y	Y	Y

*Y* yes, *N* no, *U* unclear and *NA* not applicable

**Table 2:** Characteristics of the qualitative studies selected for analysis

Author, year, country	Country	Data collection/methodology	Sample	Setting	Cause of admission (n)
Andreasen, J, et al. (2015) [30]	Denmark	Semi-structured interviews/interpretive description	7 Women 7 Men Age range: 69–93 Mean age: 80.6 years	At home approx. 1 week after discharge from hospital	4 Pneumonia 1 Emboli 1 Amputee 1 Fall 1 Brain abscess 1 Weight loss 1 Hypoglycaemia 1 Renal failure 1 Pancreatitis 1 Type 2 diabetes 1 Dizziness
Bagge et al. (2014) [41]	New Zealand	Semi-structured interviews/thematic analysis	21 Women 19 Men Age range: 75–89 Mean age: not stated	At home 1–2 weeks after discharge from hospital	Not reported
Dilworth et al. (2012) [40]	Australia	Semi-structured interview/thematic analysis	1 Woman 2 MenAge range: not stated Mean age: not stated	In hospital after being readmitted to hospital following recent discharge (within 28 days) to their homes	1 Renal failure/Fall 1 Cellulitis/Pulmonary emboli 1 Dizziness
Dossa et al. (2012) [13]	United States	Semi-structured interviews/thematic coding technique informed by grounded theory methodology	18 Men Age range:78–88 Mean age: not stated	At home 2 weeks, 1 month and 2 months after discharge from hospital	The discharge diagnoses included total hip or total knee replacements, laminectomy, diabetes, arthritis, coronary artery disease, hypertension and alcohol abuse
Jones, GB (2012) [38]	United States	Semi-structured interviews/phenomenological hermeneutical interpretation method	16 Women 4 Men Age range: 65–89 Mean age: 75 years	At follow-up visit in two cardiology/cardiovascular clinics within 4 weeks of discharge from hospital	The majority of participants had a cardiovascular medical diagnosis (*n* = 12, 60%) or experienced cardiovascular surgical procedures (*n =* 8, 40%)
Karlsson et al. (2016) [43]	Sweden	Qualitative interview/content analysis	7 Women 8 Men Age range: 65–86 Mean age: 71 years	At home within 2 months after discharge from hospital	7 Surgery of aortic aneurysm 1 Epiglottitis 1 Pneumonia, sepsis 1 Pneumonia 1 Pulmonary edema 1 Pneumonia, sepsis, kidney failure 1 Myasthenia gravis, cardiac arrest 1 Unknown 1 Allergic shock
Knight et al. (2011) [14]	United Kingdom	Semi-structured interviews/thematic analysis	4 Women 3 Men Age range: 75–91 Mean age: 82.6 years	At home 6 weeks to 3 months after discharge from hospital	Not reported
McKeown et al. (2007) [5]	Ireland	Qualitative interviews/ phenomenological approach	5 Women 6 Men Age range: 71–92 Mean age: 81 years	At home 2 weeks after discharge from hospital	Not reported
Neitherman et al. (2015) [15]	Canada	Semi-structured interviews/thematic analysis	7 Women 10 Men Age range: 70–89 Mean age: 79 years	At home 2–5 weeks after discharge from hospital	The most common diagnoses for hospitalisation were cardiovascular conditions (congestive heart failure, stroke) and respiratory problems (chronic obstructive pulmonary disease, pneumonia). Other patients had a variety of health problems, including diabetes, kidney disease, gastro-intestinal and neurological problems and cancer
Perry et al. (2011) [11]	New Zealand	Semi-structured interviews/interpretative phenomenological analysis	8 Women 3 Men Age range: 66–88 Mean age 76.3 years	At home approx. 6 weeks after discharge from hospital	Orthopaedic lower limb surgery
Reay et al. (2015) [39]	Australia	Semi-structured interviews /Giorgi’s phenomenological method	6 Women 4 Men Age range: not stated Mean age: not stated	At home approx. 3 weeks after discharge	Total hip replacement surgery
Rydeman et al. (2008) [42]	Sweden	Semi-structured interviews/grounded theory	7 Women 10 Men Age range: 65–91 Mean age: 79 years	At home 4–8 weeks after discharge from hospital	4 Infection 4 Heart problems 1 Rheumatic disease 3 Intestinal problems 1 Dehydration 1 Fracture 1 Pneumonia stroke 1 Intoxication
Slatyer et al. (2013) [37]	Australia	Semi-structured interviews/thematic content analysis	6 Women 6 Men Age range: 72–91 Mean age: 81.6 years	At home within 28 days of discharge (after readmission to hospital)	Breathing, gastric, renal or cardiovascular problems; falls; or chest pain7

The samples for the individual studies ranged from 3 to 40 participants, each over 65 years old. The collective sample represented the experiences of 195 older adults, 95 women and 100 men. The 13 studies were conducted in 8 different countries. Eleven of the studies were conducted in the participants’ homes after hospital discharge, one at a hospital follow-up visit within four weeks of discharge to home and one at the hospital after being readmitted to hospital following recent discharge to home. These studies addressed the clearly stated aims of the research, the data analysis was easy to follow, and the results were unambiguous throughout. The participants’ voices were easily identifiable and separate from the researchers’ own interpretations of the results. Furthermore, almost all of the studies included meaningful considerations of the relationship between the researcher and participants, and all addressed ethical issues.

The extraction phase resulted in 42 thematic statements (Table 3). During the abstraction phase, these themes were merged in order to capture the content of the findings accurately. Four main themes emerged from the material: (1) Experiencing an insecure and unsafe transition, (2) settling into a new situation at home, (3) what would I do without my informal caregiver? and (4) experience of a paternalistic medical model. Examples of participants’ quotations illustrating these results are presented in (Additional file 1:Table S1).

**Table 3:** Findings, including main themes and thematic statements, with calculated frequency effect % (rounded to nearest whole number)

Findings	Included studies	Frequency effect %
Theme 1: Experiencing an Insecure and Unsafe Transition		
Lack of information about health situation, treatment and/or care	[11, 13, 14, 30, 37, 38, 40–42]	69%
Experience of rushed discharge	[14, 15, 37, 38, 40–43]	62%
Confusion about medication	[5, 14, 15, 30, 38, 40–42]	62%
Lack of involvement in own treatment and care	[11, 13, 14, 37, 40–42]	54%
Not being involved in decisions about own life	[11, 13, 14, 37, 40–42]	54%
Not understanding information	[14, 37, 38, 40–42]	46%
Several providers coordinating care led to discontinuity of care	[13–15, 37, 38, 40]	46%
Errors in treatment	[13, 14, 30, 38, 40, 41]	46%
Discharge information not explained well	[14, 38, 40–42]	38%
Lack of information about when to go home	[11, 14, 15, 42, 43]	38%
Lack of communication between the different service providers	[13, 14, 30, 38, 40]	38%
Conflicting opinions between healthcare providers	[13, 14, 38, 40]	31%
Lack of medical reconciliation	[14, 38, 40]	23%
Experience of well-prepared and timely discharge	[30, 42, 43]	23%
Theme 2: Settling into a New Situation at Home		
Dependent on additional help from others	[5, 11, 13–15, 30, 37–43]	100%
Losing independence	[11, 13, 15, 30, 38–43]	77%
Finding the transition back home a challenge	[5, 13, 15, 30, 38–40, 43]	62%
Home not being prepared	[5, 13, 15, 30, 38–40, 42]	54%
Problems performing daily activities	[5, 11, 15, 30, 38, 39, 43]	54%
Not receiving care according to needs	[5, 15, 30, 38–40, 43]	54%
Wanting to maintain and regain independence	[11, 15, 37–39, 43]	46%
Not feeling ready to go home	[11, 37, 40, 42, 43]	38%
Feeling confident to go home	[11, 37, 39, 42, 43]	38%
Not being able to participate in meaningful activities	[5, 15, 30, 39, 43]	38%
Feeling lonely and isolated	[5, 15, 30, 39, 43]	38%
Lack of specialised equipment	[5, 13, 30, 38, 39]	38%
Changing healthcare personnel disturbed effort to get back to daily routines	[15, 30]	15%
Feeling depressed	[15, 30]	15%
Experiencing no meaning in life	[15, 30]	15%
Wanting to die	[15, 30]	15%
Theme 3: What Would I do Without My Informal Caregiver?		
Dependent on informal caregivers for medication and healthcare	[5, 14, 15, 37, 38, 40–42]	62%
Dependent on family and friends to manage daily activities at home	[5, 11, 15, 30, 37–39, 43]	62%
Being aware of the effort put in by informal caregivers	[5, 11, 30, 39, 40]	38%
Importance of strong, positive relationships with family and friends	[5, 11, 15, 30, 39]	38%
Dependent on informal caregivers to understand information	[14, 37, 41, 42]	31%
Illness putting a strain on relationship with family and friends	[11, 30, 39]	23%
Feeling like a burden	[11, 30, 39]	23%
Theme 4: Experience of a Paternalistic Model		
Healthcare personnel perceived as distant and stressed	[11, 13, 14, 30, 37, 38, 41, 42]	62%
Not being seen or heard	[11, 13, 14, 40–42]	46%
Reluctant to ask	[14, 37, 40–42]	38%
Healthcare personnel perceived as authoritarian	[11, 41–43]	31%
Doctor knows best	[14, 40–42]	31%

### Theme 1: experiencing an insecure and unsafe transition

Many of the participants’ experienced the transition home as insecure and, in some instances, unsafe and even dangerous [13–15, 30, 37–40]. This experience appears to be influenced by several factors, as reported under Theme 1 in Table 3. Several of the participants experienced a lack of information about their diagnosis, ongoing care and self-care at home, which led to feelings of anxiety and uncertainty [11, 13, 14, 30, 37, 38, 40–42]. Participants reported experiences of a rushed or poorly planned discharge, leading to information being omitted or given too hastily [14, 15, 37, 38, 40–43]. The participants had difficulties getting an overview of their medicine, as the name of the preparations and types of medicine were changed while they were in hospital [5, 14, 15, 30, 38, 40–42], and many participants said that no one talked to them about changes in their medication before discharge [14, 38, 41]. Several participants described a problem related to medication reconciliation [14, 38, 40], meaning that their prescribed medicines did not match the medicines that should have been prescribed.

The discharge process was described by many of the participants as an anxious time because they were never quite sure when they were going to be allowed home [11, 14, 15, 42, 43]. Many participants experienced a lack of shared decision-making regarding discharge and ongoing care [11, 13, 14, 37, 40–42]. They also reported not understanding parts of the information received in the hospital [14, 37, 38, 40–42] and found that discharge information was not explained to them properly or well [14, 38, 40–42]. Even when healthcare personnel took the time to explain the information, participants did not always understand the explanations and information given to them [14, 37, 41]. Healthcare professionals’ use of medical language and abbreviations, the busyness and stress of the situation and the older persons’ inability to concentrate due to their medical condition seemed to affect their understanding of their own complex healthcare situations [14, 37, 38, 40–42]. The older persons’ feelings of not being seen, heard or given an opportunity to take part in the care and planning had a negative impact on their experience of discharge and the transition to home [14, 40, 42].

Participants also described several examples of errors in the treatment that were either because of, or made worse by, poor communication between themselves and their caregivers and between healthcare providers [13, 14, 30, 38, 40, 41]. Having several caregivers responsible for organising care seemed to lead to even more confusion and discontinuity of care [13–15, 37, 38, 40], and some of the participants experienced conflicting opinions about their treatment and care between the different health professionals overseeing their case [13, 14, 38, 40]. Better communication between staff, older persons and their caregivers could, therefore, significantly improve the older persons’ experience of the discharge procedure [14, 37, 42], as described by Rydeman et al. [42]:

The participants’ individual needs were satisfied when professionals were perceived as being knowledgeable and committed in their caring functions. They gave, for instance, comprehensible and individually adjusted information, instructions and explanations regarding the disease and treatment, the likely disease progress and the discharge time scale. All written information was highly legible, e.g. typewritten with upper-case letters. The professionals showed respect, were attentive to any emotional impact, and the older persons’ and their relatives’ points of view were considered.

When these needs were satisfied, the older persons experienced a well-prepared and timely discharge, resulting in a harmonious feeling and a sense of readiness to return to daily life at home [30, 42, 43].

### Theme 2: settling into a new situation at home

Factors that influenced the experience of settling into a new situation at home are reported under Theme 2 in Table 3. Numerous studies reported that the participants were keen to return home to the security of their own environment, which was associated with recovery, independence and personal control [11, 15, 37–39, 43]. However, adaptation to daily life after discharge from the hospital was seen by many of the participants as a real challenge [5, 13, 15, 30, 38–40, 43], as cooking, dressing, bathing and other daily activities were difficult to manage immediately after discharge [5, 11, 15, 30, 38, 39, 43]. Health problems, such as tiredness, pain and lack of appetite, also caused distress [5, 30, 43].

Many participants returned to a home environment that was not ready or appropriate for their new health situation [5, 13, 15, 30, 38–40]. Environmental challenges in the home posed significant activity impediments and could result in them resorting to unsafe practices [5, 13, 30, 38, 39]. Many participants also experienced lack of specialized equipment and supplies necessary for managing at home, such as walkers, adapted toilets, shower chairs, scales, glucose meters, etc. [5, 13, 30, 38, 39]. They reported that the healthcare provided by home care was not suited to their individual needs. The participants often did not get the right type of care and/or help at the right time of day or even the right day of the week, when they needed it most [5, 15, 30, 38–40, 43]. When different people from the care services visited at unexpected times, it was disturbing to the older persons’ effort to get back to their daily routines after discharge [15, 30].

Participants also reported not being capable of participating in meaningful activities anymore, primarily due to their physical condition, leading to a more isolated social life [5, 15, 30, 39, 43]. A loss in social life created negative consequences, such as loneliness, depression, a feeling of having no one to exist for and even, for some, a wish to die [15, 30].

### Theme 3: what would I do without my informal caregiver?

Following discharge, many of the participants reported that they were dependent on additional assistance, usually provided by an informal caregiver, ranging from a spouse to an adult child, friends or neighbours [5, 11, 13–15, 30, 37–43]. The experiences related to this theme are listed under Theme 3 in Table 3. Personal networks and social support seemed to be a crucial factor for a successful recovery for most of the participants. Caregiver support included medication and care management, cooking, cleaning, dressing, shopping, transportation, personal hygiene, incision care and dressing changes and symptom management [5, 14, 15, 38–41, 43]*.* Some participants needed walkers and/or other assistance devices, and in some cases these arrangements had to be made by informal caregivers [15, 38].

Strong, positive relationships with a spouse, family, friends and/or neighbours were emphasised as being important factors in the daily life for older patients [5, 11, 15, 30, 39]. However, they were worried that their illness would put a strain on these relationships [11, 30, 39]. The participants were aware that their informal caregivers had limited time due to other commitments, and they did not want to overburden the caregivers [5, 11, 30, 39]. Several of them reported a feeling of being a burden to their closest relatives, resulting in feelings of stress, anxiety and guilt [11, 30, 39].

### Theme 4: experience of a paternalistic medical model

The paternalistic model describes the older person’s compliance with medical authority, and this was apparent in some of the participants’ experiences during their stay in hospital and under follow-up care. Factors contributing to this experience are listed under Theme 4 in Table 3. In general, the participants trusted the system, did what they were told and had no complaints [11, 37, 40, 41]. They seemed to rely on and accept the decisions and assessments made by physicians and nurses because they were regarded as being authoritarian or that ‘they know best’ [11, 40–43], making them reluctant to critically question staff about their treatment and care [14, 37, 40–42]. They experienced healthcare staff who were stressed, distant or in a hurry and did not have the time to talk to them [11, 13, 14, 30, 37, 38, 41, 42]. Some participants equated asking questions as arguing with the healthcare staff [41], and some felt they were not being heard when they questioned decisions made by the doctors [14, 38, 40, 42]. Some also felt patronised by the health professionals [11]. In situations where needs were not met after discharge, the older persons felt treated as objects and insistent and tiresome cases, and this had negative consequences for the person’s wellbeing [30, 42].

## Discussion

Our analyses of these studies indicate that during hospital discharge and transitional care, older persons commonly experience situations where healthcare professionals do not consider their need to understand and actively engage in questioning, discussion and information-seeking. This corresponds to the findings of several previous studies [12, 19–21]. The reason for physicians’ and other professionals’ neglect of the older persons’ needs in this context may be an overestimation of the person’s understanding of the post-discharge treatment plan and assumption that a person knows more about treatment and recovery than they actually do [44]. This emphasises the need for improved communication between professionals and older persons, as well as giving older persons the opportunity to be involved in decision making regarding their own health, to be standard practice.

Previous research shows that improved doctor–patient communication and patient participation can increase patient and provider satisfaction, as well as improve the patient’s management of their chronic illness (es) [45]. Studies have demonstrated a correlation between effective physician–patient communication and improved health outcomes [46]. At the healthcare-system level, patient participation may potentially reduce healthcare costs [47], as well as medical errors [48]. Kristiansen et al. [49] found that being in control, experiencing a sense of power, feeling trust and being given the opportunity to participate was important for older persons’ satisfaction with their care services.

All of this corresponds with strategies for increased user involvement that have appeared in the policy and action agendas of healthcare providers in recent years [50, 51]. However, older persons may find it a challenge to be involved and participate actively in discharge planning because they find it difficult to understand what is being discussed or fail to feel included in the conversation [20]. To be able to involve older persons in the decision making, it is important that verbal and written communication and information is clear and easy to understand. Healthcare personnel need to take into account that the geriatric population are at risk of inadequate or marginal health literacy [52], and they should assess whether the information is understood by, and even understandable to, the person [53]. In addition, cognitive impairments or physical disabilities may interfere with older persons’ ability to be their own advocates. Hence, healthcare delivery systems need to be carefully thought out in order to appropriately support these persons [54]. Some persons may prefer to have a limited involvement in decision making [55, 56], but in this case, it is important for healthcare providers to attempt to clarify the reason for this and try to find and encourage a level of involvement that is satisfactory to the patient.

Furthermore, a study by Richardson et al. [57] found that persons aged 80 and over were reluctant to say or ask anything that could be perceived as criticising or complaining about the hospital or the hospital staff. This may be explained by the fact that this generation of older people has often had a lifelong experience with a paternalistic healthcare system [58], which aligns with our findings. To reduce the feelings of disempowerment and improve their experience of the transitional process, better bidirectional communication and information is needed. Professionals will need to modify and/or bolster their interviewing skills, as well as provided oral and written information, and spend additional time getting to know the older person and determine their values, goals and preferences [21].

Our findings are in line with previous studies that report that healthcare sectors experience difficulties in communication and systematic information exchange, as well as with coordinating, which could lead to adverse events, such as readmissions, drug events, and falls [26]. The multifaceted arrangements of multiple service providers and the complexity of the needs of older patients make coordination of care challenging [59].

The participants in these studies were generally eager to go home and, they stressed their ability to manage on their own at home after discharge. This corresponds to a previous study by Ebrahimi et al. [60], which found that older persons made great efforts to find ways to master life’s new situation, be useful and not be a burden to others. However, inadequate assessment of personal needs evoked feelings of frustration and led to limited social interactions, social isolation and loneliness. The findings indicate that when environmental and psychosocial needs are unaddressed, it affects self-management and recovery at home after discharge. Involving informal caregivers in the discharge process and the assessment of the older persons’ needs at home can give older people better opportunities to master life at home after hospital discharge, thus reducing the strain on family caregivers. The systematic review by Bauer et al. [29] revealed that conducting comprehensive discharge planning that includes the older person and their informal caregivers is directly related to a reduction in hospital readmission, shorter hospital stays and improved satisfaction with the healthcare experience.

### Implications for practice

Given that most healthcare systems are moving towards a model that favours early discharge from hospital to home or community care, it is imperative to understand how care providers can improve continuity of care to make the transitional process smoother for older persons. Strategies should include:

- Older persons receiving (sufficient) information about their illness (es) and the course of the illness, medication, rehabilitation and psychosocial aspects of their recovery.
- Hospital discharge should include an assessment of the person’s post-discharge needs when it comes to food, specialised equipment, transportation to follow-up appointments and general care at home. These identified needs should be communicated to the next level of care.
- Adapting verbal and written communication in order to make the information clearer and easy to understand. Healthcare professionals should also assess whether the information is understood by the patient.
- Incorporating older persons’ involvement and perspectives into the decision-making process.
- Increasing the involvement of informal caregivers in the care planning.
- Strategies to reduce feelings of loneliness and social isolation after hospital discharge and resettling into the home situation.
- Better communication between service providers, professionals, older persons and their informal caregivers.

### Strengths and limitations of the study

A major strength of this work is the application of a rigorous and systematic metasummary technique. Synthesising qualitative research is viewed as essential to achieving the goal of evidence-based practice, namely to use the best available evidence as the foundation for practice without methodological prejudice [31]. Another strength is that even though the included studies are from different countries with variously structured healthcare systems, there was considerable agreement across the research about how older persons experienced hospital discharge and adapting to daily life at home afterwards.

Because care models are continuously reformed—for example, the Affordable Care Act in the United States [61], evolving models of ambulatory and sub-acute care funded by the Australian government [9] and evolving models in the British Healthcare Trusts [62]—we only included studies published between 2006 and 2017 to ensure the findings would be relevant to current clinical practice. These studies are grounded in policy and practice contexts of the more recent transitional care and integrated care models in the countries represented. However, by excluding earlier studies, our study may not address issues pertaining to user experience in discharge and care transitions from preceding healthcare arrangements. We also only included studies published in English in our review; therefore, we may have missed relevant research from non-English-speaking countries.

## Conclusions

This study contributes to our understanding of older persons’ experiences of the transition from hospital to home and may provide an important frame for understanding and improving older persons’ satisfaction with the healthcare services provided in transitional care. The results emphasise the importance of assessment and planning, information and education, preparation of the home environment, the involvement of the older person and caregivers and supporting self-management in the discharge and follow-up care at home. Health professionals should actively cultivate their communication skills with an awareness of older persons’ experiences, priorities and goals in order to provide healthcare focused on what is most relevant to older persons in transitional care situations. Better communication between older persons, hospital providers and home care providers is needed to improve coordination of care and facilitate recovery at home. The organisational structure may need to be redefined and reorganised to secure continuity of care and wellbeing of older persons in transitional care situations.

## Additional file

Additional file 1: **Table S1.** Examples of participants’ quotations illustrating the results. (PDF 103 kb)

## Abbreviations

HRQOL: Health-Related Quality of Life
JBI-QARI: Johanna Briggs Institute Qualitative Assessment and Review Instrument
