# Supplementary material for: Older persons’ experiences of adapting to daily life at home after hospital discharge: a qualitative metasummary
Source: BMC Health Serv Res. 2019 Apr 11;19:224. doi: 10.1186/s12913-019-4035-z (PMC6460679; doi:10.1186/s12913-019-4035-z)
Supplement: Supplementary file 1 — Table S1. Examples of participants’ quotations illustrating the results. (PDF 103 kb) [file 12913_2019_4035_MOESM1_ESM.pdf]

Additional file 1

Table 4: Examples of participants' quotations illustrating the results.

Theme 1: Experiencing an Insecure and Unsafe Transition	
Lack of information about health situation, treatment and/or care	'They haven't told me ANYTHING was the matter with me as far as I know. No, I said, WHAT'S THE MATTER WITH ME? I know I am dumb, but I am sure they could explain it in layman's terms. It's all I want to know, what is the matter with me? Why am I getting these dizzy spells and what is causing them? I don't think it is too hard to answer' [40].<br><br>'There was a dearth of information about what to expect, in terms of progress…I would like to have had some encouraging picture of the future…how progress would be, and various milestones, and right timings' [11].<br><br>'That was a bit of an issue really…there was no card to give any explanation and no nurses gave any explanation about it' [14].
Lack of involvement in own treatment and care	'What one is that? You see this is another thing, nothing's been explained to me! I've never seen that one before in my life! It was in the bag when I came out of hospital!' [14]<br><br>'I asked [questions] because I didn't feel well when being discharged, but the doctor told me that there was no more to be done at hospital. Next day, I was readmitted' [42].<br><br>'I think we were all kicked out [of hospital]. Because it was a long weekend' [11].<br><br>'They just needed the bed. They were short of beds. I wasn't a serious case…more serious cases [were] waiting to occupy a bed' [37].<br><br>'But we tried to get a visiting nurse to remove the stitches. I kept calling. I had two nurses and one therapist calling constantly. They wouldn't return the phone calls, and we tried to get permission from them to let the stitches be removed here, rather than make a round trip to [the hospital] for a 2 minute procedure, you know? And they said, "Oh no, the doctor wants to see you." So, I went to the hospital and he got the stitches pulled and I didn't even go to the same hospital. And I thought, *Well, the doctor will be there*. There was some doctor I never heard of there. Then what was the point of me going—You know, I could have had it done right here at this VA or by my visiting nurse' [13].<br><br>'No, only the doctor just mentioned this afternoon, just before you arrived, he said we're thinking about sending you home, I said,

	"beauty." That was that, he just examined me and ducked off' [40].
Errors in treatment	'Well, I am a bit, you know, I am bit annoyed about the anti-inflammatory I was given, because I told the doctor no, no, no, no, no I can't have them and he put—he gave me some, see I wasn't aware the nurse comes along and there are loads of little tablets and they're all different colours from what I am used to, you know, and, um, I didn't, wasn't aware but she did say... I am sure she said "indocid" and thought oh she couldn't have said that because I am not having that. Anyway, so I swallowed down the tablets, well then…I got kidney failure so, yeah, and that's why I've got this (indicates tubes inserted into neck above clavicle) this has to, oh it's a horrible thing it's, um, the dialysis, it must be like an enormous cannula or something and, um, so now, oh, and I hope it will only be temporary, because otherwise it's dialysis for the rest of my life and that is terrible, I am just so annoyed' [40].
Lack of communication between service providers	'It seems rather ridiculous, but they just don't deal with each other. And don't even agree with each other. One will prescribe one medicine and they'll prescribe another. And all they say is, "(PCP) will bow to (surgical site)," and say, "Okay, do whatever they say." There's no communication here—I don't know what the problem is. They don't work close, they work independent of each other' [13].
Lack of medical reconciliation	'We ran into a problem last night, where we got some medication. Some [printed instructions] say don't take and others saying take. I don't know what the hell I'm supposed to do. Why would one group tell me to take it and another group tell me not to take it?' [38]
**Theme 2: Settling into a New Situation at Home**	
Losing independence	'So I am anxious about whether I will be locked up in this house or I may happily be able to use prostheses, and for God's sake please do not lock me up in here, but give me a handicap vehicle, so that I can get around' [30].<br><br>'Oh, I did so much: I sewed, I went to different centres and learned so many things, well yes, I have been active. I also went swimming and I would have liked to continue that, but I couldn't due to the long distance to the swimming pool, so I can't walk down there' [30].
Home not being prepared	'I came home to a house where nothing was taken care of. Nothing! There was a "bedpan chair" with no bedpan beneath, so I could just sh*t on the floor. That was it!' [30]<br><br>'They [hospital] had a lady who drove the car and she brought me here [home]…Everything was alright…except that I did not have

	the food in my fridge because I emptied the fridge before the hospital because I knew that I would be so long so the food would get spoiled' [15].
Not receiving care according to needs	'Horrible, horrible, horrible [to] come to an empty house and have to manage all [by] yourself after four days. I was very upset. It's too much for old people. I have no family and these people upstairs, they never knock on the door' [39].<br><br>'It was difficult the first few weeks…it was, you felt you had nobody to help you, you were just on your own. My husband, he's got Alzheimer's and he can't do much…There's a lot to think about and a lot to do when you come [home], especially in my case having me back [spinal stenosis] as well, I've got the two, if I hadn't got me back my hip would be alright. I didn't go to bed for three nights, I couldn't go, couldn't go, couldn't get in and out of bed, just sat in the chair' [39].
Wanting to maintain and regain independence	'Not being able to get out, frustrated by not being able to drive, having to rely on others who lead busy lives. I'm very independent and don't like relying on others' [39].
Feeling lonely and isolated	'Loneliness when I came home…I'm more often than not without anyone…Out of this room, it's terrible 'cause I was so full of going…At times now when the helper would be gone…[I'm] so lonely I'd go to sleep…I love someone to have a little chat with, you kind of need that because loneliness is worse than hunger' [5].
Experiencing no meaning in life	'Nothing is important anymore. No, there is no meaning to anything. Sometimes I want to die, because there is no meaning to anything. The kids have grown old and the grandchildren and all that, so I have experienced that' [30].
**Theme 3: What Would I do Without My Informal Caregiver?**	
Dependent on family and friends to manage daily activities at home	'Whatever I want she [home help] does, the shopping, washing, the ironing, all that has to be done in a house 'cause I can't do [it]…I'd need someone of them [family] have to be around on a Saturday and Sunday. I'd have no one to give me breakfast in the morning' [5].
Being aware of the effort put in by informal caregivers	'…all the time she takes care for me, for my food, for everything that I eat, and she takes care for clean my stuff [dressing change to incision].' He further explained the frequency with which his wife changed his wound dressing. He explained that '…she takes care for every 4 hours [to] change dressing' [38].<br><br>'If I can't manage or the girls [daughters] can't manage, as they're all working themselves and they can't give up…I can't expect them to give up their little holidays…they have their family and they can't deprive their family' [5].

Importance of strong, positive relationships with family and friends	‘I found it awful, that is for sure. And did I not have such good neighbours, I would never have succeeded, never’ [30].<br><br>‘What is really important for me is, in fact, that our marriage is functioning and I think it still does, but I have, in fact, been nervous that it maybe would break down, because this has been too big a strain’ [30].<br><br>‘My neighbour, she’s good, she will take me anywhere, she paid my bills yesterday, she comes up twice to feed the chooks. I’m pretty lucky because the people around me are good, that’s where I’m thankful if I did want something, someone would help’ [39].
**Theme 4: Experience of a Paternalistic Medical Model**	
Healthcare personnel perceived as distant and stressed	‘…a doctor I’d never seen before wanted to look at the scar, so I take my pants down for this strange man. He looked at the scar and he said how good it was; I said, “yes, my ankles are more [of a] problem than the hip.” He said, “Well we’re only looking at the hip…right, well, we’ll see you in a year.” We didn’t sit down even…but I mean, they’re all so terribly busy’ [11].<br><br>‘Well, they say very little, they just say you’re on this and this and this, do you understand and they’re keen to get off’ [14].<br><br>‘I wouldn’t even [know] how to say “why am I taking these pills?” because I might be disregarding their, you know, position or something’ [41].
Healthcare personnel seen as authoritarian	‘[The surgeon] cleared me—last week…doesn’t want to see me till October this year, which is about ten months…said that I was a “good boy, good patient”’ [11].
Doctor knows best	‘Because he is a specialist advising, I’m taking their advice, I’m not going to argue with them about it’ [41].
